# Photoinhibition-Like Damage to the Photosynthetic Apparatus in Plant Leaves Induced by Submergence Treatment in the Dark

**DOI:** 10.1371/journal.pone.0089067

**Published:** 2014-02-19

**Authors:** Xingli Fan, Zishan Zhang, Huiyuan Gao, Cheng Yang, Meijun Liu, Yuting Li, Pengmin Li

**Affiliations:** 1 State Key Lab of Crop Biology, College of Life Sciences, Shandong Agricultural University, Tai’an, Shandong, China; 2 State Key Laboratory of Crop Stress Biology for Arid Areas, College of Horticulture, Northwest A&F University, Yangling, Shanxi, China; US Naval Reseach Laboratory, United States of America

## Abstract

Submergence is a common type of environmental stress for plants. It hampers survival and decreases crop yield, mainly by inhibiting plant photosynthesis. The inhibition of photosynthesis and photochemical efficiency by submergence is primarily due to leaf senescence and excess excitation energy, caused by signals from hypoxic roots and inhibition of gas exchange, respectively. However, the influence of mere leaf-submergence on the photosynthetic apparatus is currently unknown. Therefore, we studied the photosynthetic apparatus in detached leaves from four plant species under dark-submergence treatment (DST), without influence from roots and light. Results showed that the donor and acceptor sides, the reaction center of photosystem II (PSII) and photosystem I (PSI) in leaves were significantly damaged after 36 h of DST. This is a photoinhibition-like phenomenon similar to the photoinhibition induced by high light, as further indicated by the degradation of PsaA and D1, the core proteins of PSI and PSII. In contrast to previous research, the chlorophyll content remained unchanged and the H_2_O_2_ concentration did not increase in the leaves, implying that the damage to the photosynthetic apparatus was not caused by senescence or over-accumulation of reactive oxygen species (ROS). DST-induced damage to the photosynthetic apparatus was aggravated by increasing treatment temperature. This type of damage also occurred in the anaerobic environment (N_2_) without water, and could be eliminated or restored by supplying air to the water during or after DST. Our results demonstrate that DST-induced damage was caused by the hypoxic environment. The mechanism by which DST induces the photoinhibition-like damage is discussed below.

## Introduction

Submergence is one of the common environmental challenges for plants in natural ecosystems [1], [2]. It hampers growth and survival of plants and causes a significant decrease in crop yield, mainly by inhibiting plant photosynthesis [1], [4]–[6].

Most previous studies show that the photosynthesis rate and photochemical efficiency decrease in the leaves of submerged plants [3], [7], [8]. The decline in activity of the photosynthetic apparatus is induced, firstly, by the inhibition of root respiration by low O_2_ levels in submerged soil, which decreases the absorptive capacity for water and nutrients [1], [9], [10]. Secondly, signals such as abscisic acid (ABA) and ethylene from the hypoxic roots lead to stomatal closure and leaf senescence, thereby inhibiting photosynthesis and causing photoinhibition [7], [11], [12]. Thirdly, the low CO_2_ concentration in water inhibits photosynthetic carbon assimilation, triggering excess excitation energy [13]–[15].

Signals from hypoxic roots and light-induced excess excitation energy are harmful to the photosynthetic apparatus. However, it is unclear whether mere leaf submergence affects photosynthetic activity without the influence of the factors mentioned above.

To address this question, we explored the function of the photosynthetic apparatus in detached leaves submerged in the dark (dark-submergence treatment, DST), which is a method of avoiding the influence of excess excitation energy and signals from the roots to the photosynthetic apparatus.

## Materials and Methods

### Plant Materials

The leaves used in this study were detached from four plant species, grown on an experimental plot of land owned by our University. No specific permissions were required for using plant materials from our experimental land. Our field studies did not involve endangered or protected species. The specific coordinates of the area are 36 degrees north and 117 degrees east.

We used the youngest fully expanded leaves of four plant species: daylily (*Hemerocallis fulva*), willow (*Salix babylonica*), euonymus japonicus (*Buxus megistophylla Lévl*) and maize (*Zea mays L*.). The leaves were detached before sunrise from plants naturally grown in the field and taken to the laboratory wrapped in damp cloths as soon as they were detached.

### Dark-submergence Treatment

Leaves were wrapped in a damp cloth and placed in a completely dark environment (control, CK) or fully submerged into deionized water in the dark (dark-submergence treatment, DST) at different temperatures (15°C, 25°C, and 35°C). The temperature was controlled by a GXZ incubator (Ningbo, China). Parameters such as Fv/Fm, Ψo, ABS/CSm and Activitiy of PSI complex were measured after 0 h, 12 h, 24 h and 36 h of treatment.

To elucidate the influence of O_2_ on the photochemical activity of leaves during submergence, air was continually pumped into the water at a 0.17∼0.2 m^3^·s^−1^ flow rate during DST (DST+Air). To explore the recovery of the photochemical activity of leaves after DST, with or without O_2_, air or N_2_ at a 0.17∼0.2 m^3^·s^−1^ flow rate was pumped into the water for 36 h after 36 h of DST. To exclude the physical influence of water, the detached leaf segments wrapped in damp cloths were placed in the air (CK) or N_2_ environment.

### Measurements of Chlorophyll a Fluorescence Transient and Reflection of 820 nm Modulated Light

The chlorophyll a fluorescence transient and the reflection of 820 nm modulated light changes were simultaneously measured using an m-PEA (Hansatech, UK). The saturating red light of 5000 µmol m^−2^ s^−1^ was produced by an array of four light-emitting diodes (LED, peak 650 nm). All the measurements were performed with dark-adapted leaf at room temperature. The chlorophyll a fluorescence transients were obtained by 2 s saturating red light and analyzed with the JIP-test [16]. The description and calculation of standardization formula of OJIP transients and formula of parameters were listed below.

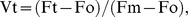
 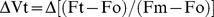

2. Maximum quantum yield of PSII, 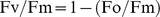

3. Efficiency of electron moves beyond Q_A_^−^, 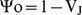

4. The density of Q_A_-reducing PSII reaction centers per cross section,

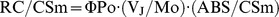

5. The normalized relative variable fluorescence at the K step (W_K_), 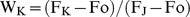

The modulated measuring light (820 nm) was provided by an m-PEA. Irradiated with a saturating red light (5000 µmol m^−2^ s^−1^ PFD), the reflection at 820 nm in leaves decreases gradually, which is mainly caused by the initial oxidation of P700 (the primary electron donor in PSI) and plastocyanin (PC) [17]. The initial slope of the reflection at 820 nm light namely the activity of PSI complex was used to reflect the PSI activity [18], [19].

### Measurement of the Oxygen Evolution Rate

An OXYTHERM oxygen electrode (Hansatech, UK) was used to measure the O_2_ evolution rate of leaves in 50 mM NaHCO_3_ solution (dissolved in 50 mM Tris-HCl buffer, pH 7.5) at 25°C. A photosynthetic saturation light (1600 µmol m^−2^ s^−1^) was used in the measurements.

### Detection of D1 and PsaA Protein

The protein was detected with thylakoid membranes of the treated leaves by Western Blot. For thylakoid membranes preparation, leaf fragments were homogenized in an ice-cold isolation buffer (100 mM sucrose, 50 mM Hepes, pH 7.8, 20 mM NaCl, 2 mM EDTA, and 2 mM MgCl_2_) and then filtered through three layers of pledget. The filtrate was centrifuged at 3000 g for 10 min. The sediments were washed with isolation buffer, re-centrifuged, and then finally suspended in an isolation buffer. The thylakoid membrane proteins were then denatured and separated using 12% polyacrylamide gradient gel. The denatured protein complexes in the gel were then electro-blotted to PVDF membranes, probed with D1 or PsaA antibody, and then visualized by the enhanced chemiluminescence method. The quantitative image analysis of protein levels was performed with Gel-Pro Analyzer 4.0 software.

### High Light Treatment

In high light treatment, leaves were illuminated under 1000 µmol m^−2^ s^−1^ light provided by red and blue (8∶1) light emitting diode light source (LED; Senpro, China).

### Measurement of the Chlorophyll Content

Leaf chlorophyll was extracted with 80% acetone in the dark for 72 h at 4°C. The extracts were analyzed using an UV-visible spectrophotometer UV-1601 (Shimadzu, Japan) according to the method of Porra [20].

### Measurement of the Hydrogen Peroxide Content

Tissue hydrogen peroxide content was estimated according to Brennan and Frenkel [21]. H_2_O_2_ content was calculated from a standard curve prepared by using different concentrations of H_2_O_2_ solutions (75–750 nmol mL^−1^ working solutions prepared from a 1 mM stock solution).

### Measurements of the O_2_ Concentration of the Water

The O_2_ concentration of the water during the dark-submergence treatment was measured by OXYTHERM oxygen electrode (Hansatech, UK) at 25°C which was automatically controlled by OXYTHERM. Two mL water was added to reaction vessel, the O_2_ concentration (nmol mL^−1^) was recorded after the signal of O_2_ reaches a steady state.

### Statistical Analysis

Calculations of standard error (SE) were carried out with Microsoft Excel software. Least significant difference (LSD) was used to analyze differences between the different treatments by using SPSS 16.

## Results

### Changes in the Photoactivities of Photosystem I and Photosystem II during Dark-submergence Treatment

**Figure 1:**
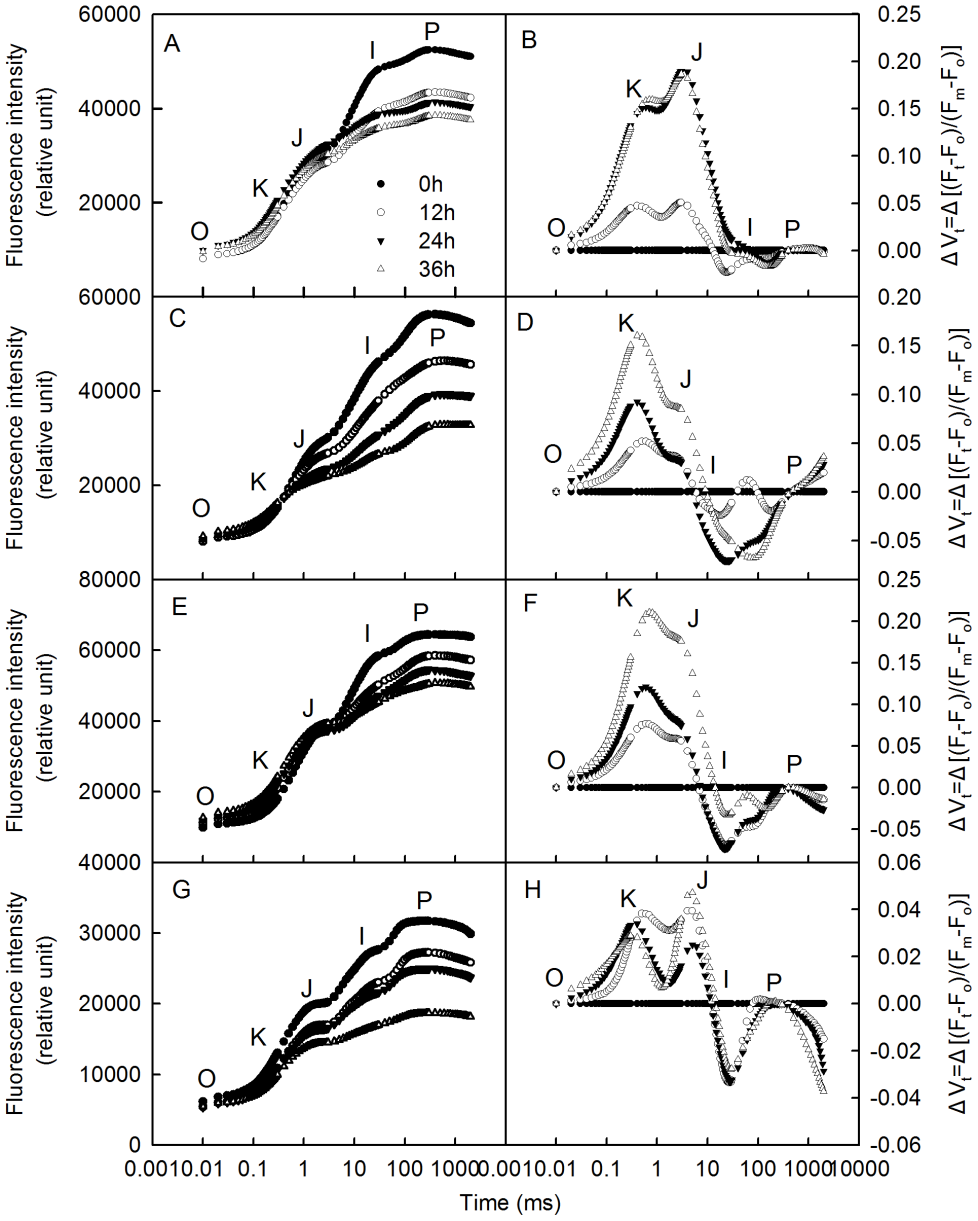
Chlorophyll a fluorescence transients and ΔVt curves in leaves during dark-submergence treatment. Chlorophyll a fluorescence transients (A, C, E, G) and ΔVt curves (B, D, F, H). A, B = daylily; C, D = willow; E, F = euonymus japonicus; G, H = maize. Vt is the standardization from the O to P step 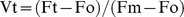. ΔVt was obtained by subtracting the kinetics of leaf segments before from those after treatment. Each transient is the mean of eight replicates.

**Figure 2:**
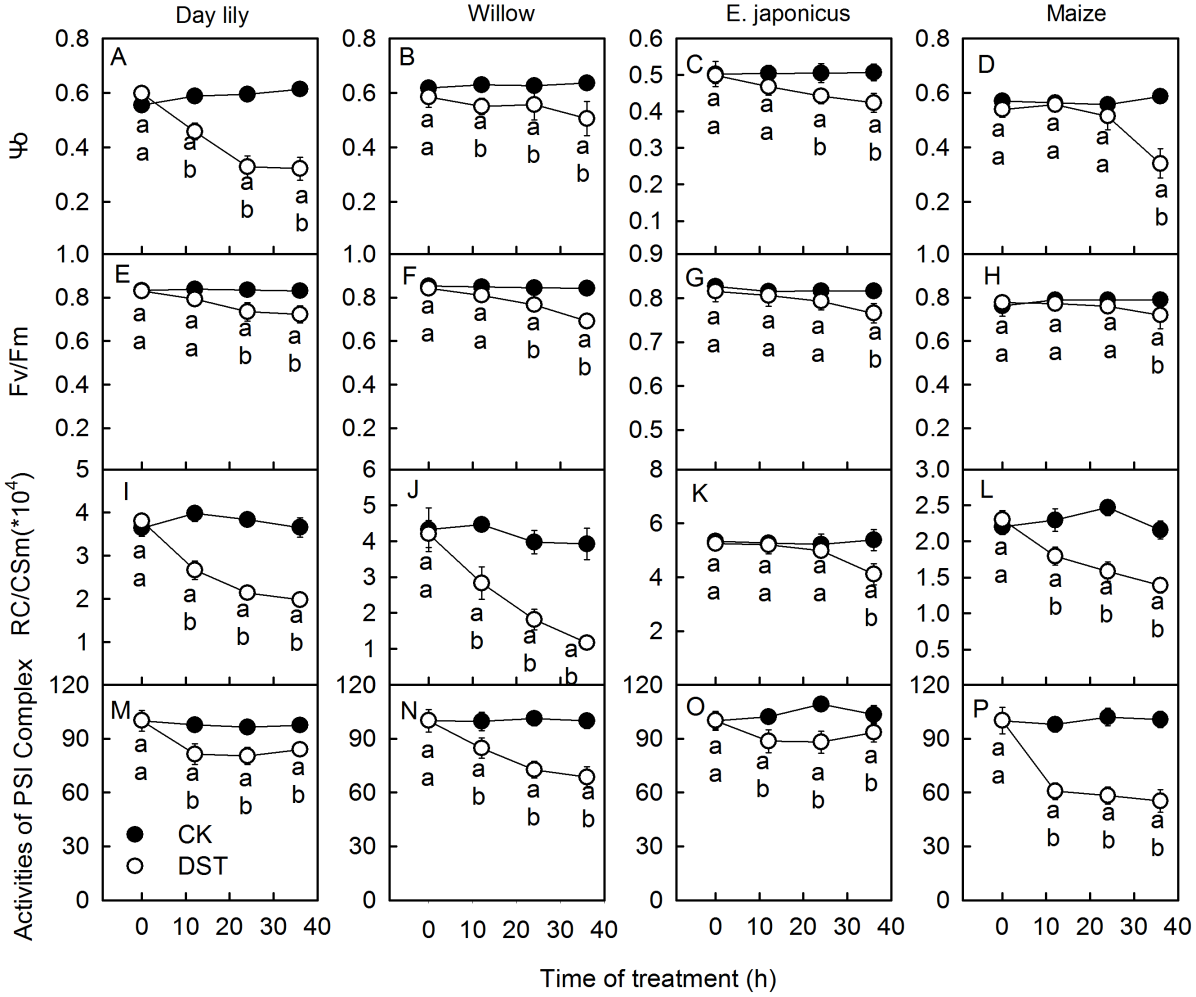
PSII and PSI activity in leaves during dark-submergence treatment. The efficiency of electron moves beyond Q_A_^−^ (Ψo), maximum quantum yield of PSII (Fv/Fm), density of Q_A_-reducing PSII reaction centers per cross section (RC/CSm) and activity of PSI complex in daylily (A, E, I, M), willow (B, F, G, H), euonymus (E.) japonicus (C, G, K, O) and maize (D, H, L, P) leaves during dark-submergence treatment (DST) were obtained by the JIP-test and the analysis of 820 nm light reflection. Means ± SE of eight replicates are presented. Different letters indicate significant differences between the treatments, P<0.05. Differences were analyzed by least significant difference (LSD).

Chlorophyll a fluorescence transients (OJIP) containing abundant information about the primary photochemical reactions of PSII, have been widely used in PSII activity studies [22], [23]. The appearance of the peak at J step (at 2 ms) and the decrease in efficiency of electron moves beyond Q_A_^−^ (Ψo) indicate that the PSII acceptor side is inhibited. More specifically, the electron moves beyond Q_A_^−^ is limited [24], [25], [26]. The J step (at 2 ms) in ΔVt curves increased (Fig. 1) and the Ψo decreased significantly after DST in all four plant species (Fig. 2), which indicates that the acceptor side activity was inhibited during DST.

**Figure 3:**
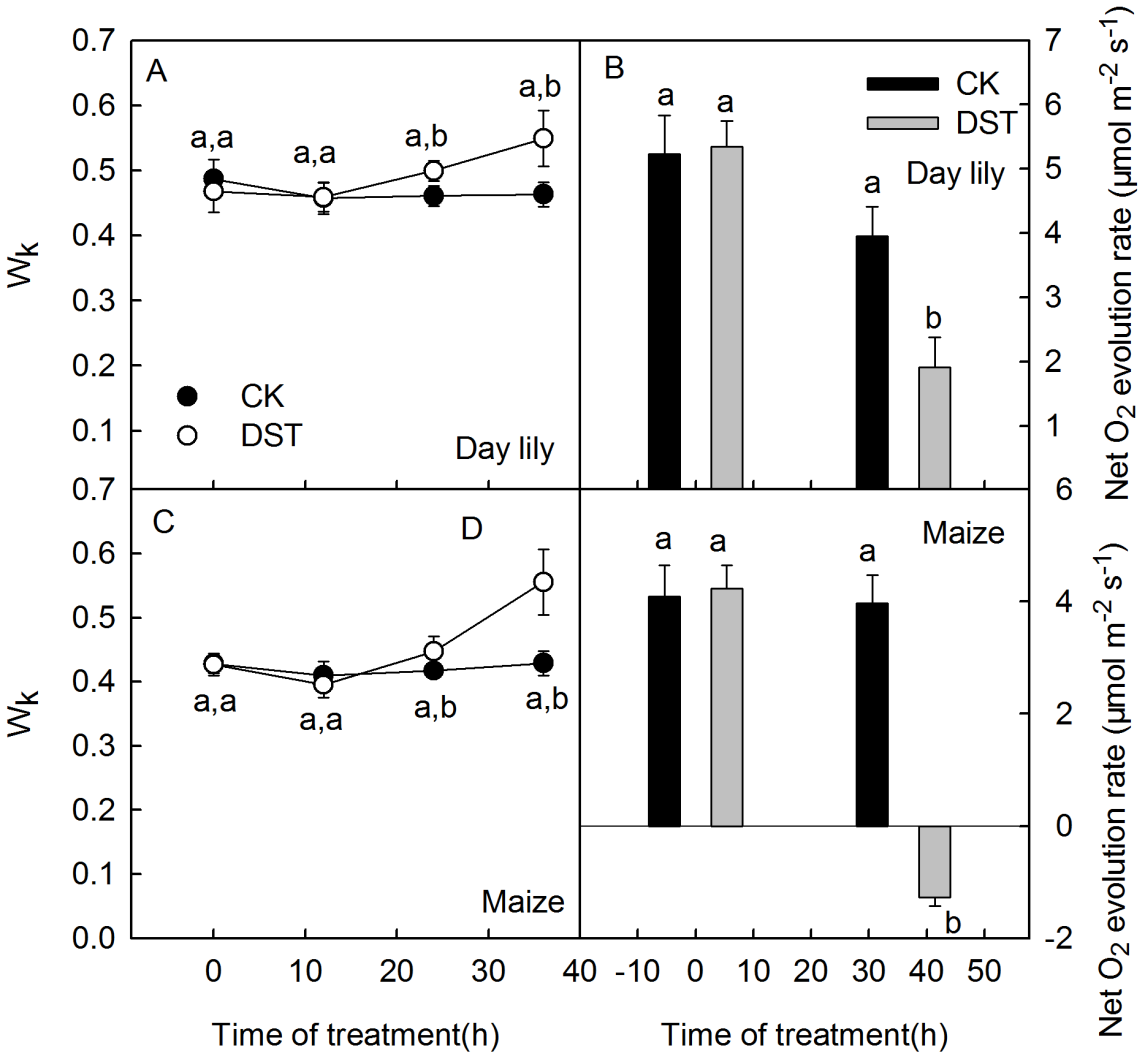
W_k_ and net O_2_ evolution rate in leaves during dark-submergence treatment. The relatively variable fluorescence at the K step (W_K_) and net O_2_ evolution rate in the daylily (A, B) and maize (C, D) leaves during dark-submergence treatment (DST). Means ± SE of eight replicates are presented. Different letters indicate significant differences between the treatments, P<0.05. Differences were analyzed by least significant difference (LSD).

Increases in K steps (at 300 µs) in ΔVt curves and W_K_ are widely used as specific indicators of injury to the donor side of PSII [24], [27], [28]. To further investigate the activities of PSI and PSII during DST, we measured the net O_2_ evolution rate and analyzed the levels of the PSI and PSII core proteins, D1 and PsaA, using two representative plant leaves (daylily (C3) and maize (C4)). The K step of the OJIP transients increased after 36 h of DST (Fig. 1), and W_K_ changed similarly to the K step (Fig. 3A, C). In addition, the net O_2_ evolution rate also decreased after DST (Fig. 3B, D). These results indicate that the donor side activity of PSII was inhibited during DST.

The maximum quantum yield of PSII (Fv/Fm) (Fig. 2) and the density of Q_A_-reducing PSII reaction centers per cross section (RC/CSm) (Fig. 2) in the leaves of different species dramatically decreased during DST. This indicates that the reaction centers in the leaves were damaged during DST.

The decrease of Fv/Fm, RC/CSm and Ψo are conventional indicators of photoinhibition induced by high light [29], [30], [31]. However, the damage occurred in leaves without light, and is therefore referred to as a “photoinhibition-like phenomenon”.

**Figure 4:**
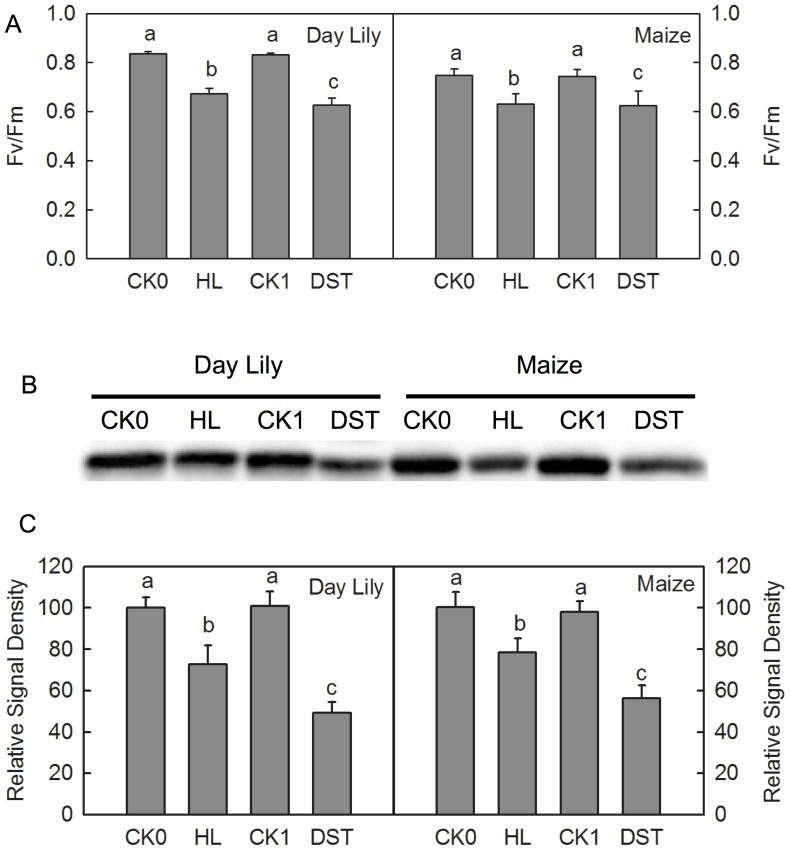
Photochemical efficiency and D1 protein levels in leaves after high- light and dark-submergence treatment. Photochemical efficiency(Fv/Fm)and D1 protein levels in daylily and maize leaves after high light (HL) and dark-submergence Treatment (DST). (A) Changes of photochemical efficiency of daylily and maize leaves. CK0, the control leaves placed in the humid-dark air for 3 h; HL, leaves after high-light (1000 µmol m^−2^ s^−1^) treatment for 3 h; CK1, the control leaves placed in the humid-dark air for 36 h; DST, leaves after DST for 36 h. Means ± SE of eight replicates are presented. Different letters indicate significant differences between different treatments, P<0.05. Differences were analyzed by LSD (least significant difference). (B) Changes in D1 protein levels after high light (HL) and dark-submergence treatment (DST). (C) Quantitative image analysis of protein levels for Fig. 9B. Means ± SE of three replicates are presented. Different letters indicate significant differences between the treatments, P<0.05. Differences were analyzed by least significant difference (LSD).

The high-light-induced photoinhibition is mainly due to the net degradation of the D1 protein [29]. To investigate whether the photoinhibition-like damage caused by DST also resulted from the degradation of the D1 protein, we performed a Western Blot analysis of thylakoid membrane preparations of leaves with both high light treatment and DST, with equal amounts of chlorophyll. The results showed that both treatments significantly decreased D1 levels (Fig. 4). This indicates that the D1 degradation was also involved in photoinhibition-like damage.

**Figure 5:**
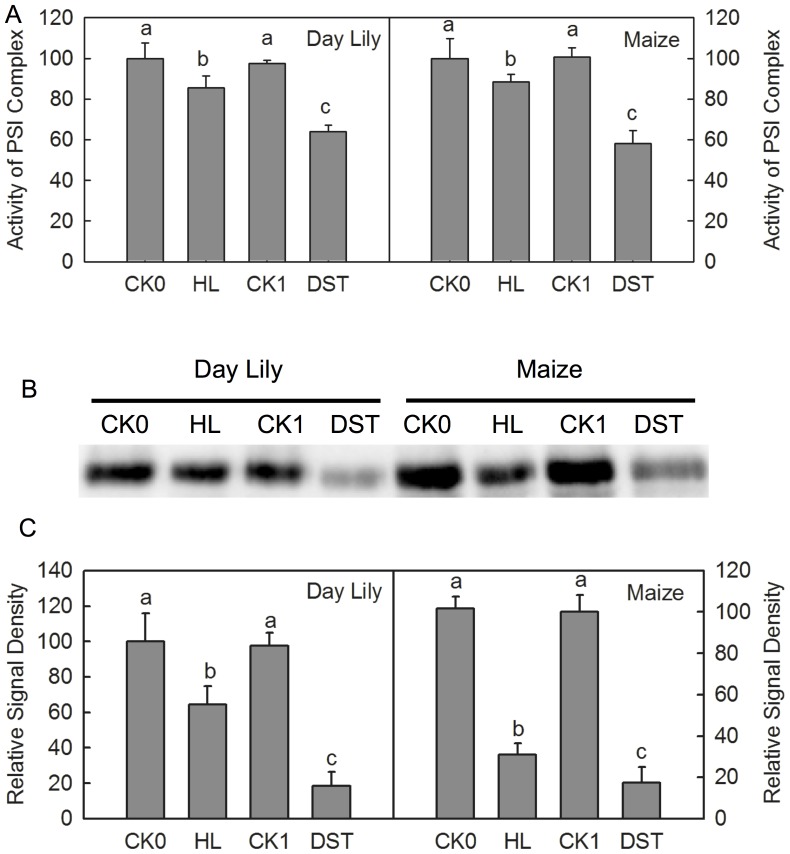
PSI activity and PsaA protein levels in leaves after high light and dark-submergence treatment. PSI activity (Activity of PSI Complex) and PsaA protein levels in daylily and maize leaves after high light (HL) and dark-submergence treatment (DST). (A) Changes of photochemical efficiency in daylily and maize leaves. CK0, the control leaves placed in the humid-dark air for 3 h; HL, leaves after high light (1000 µmol m^−2^ s^−1^) treatment for 3 h; CK1, the control leaves placed in the humid-dark air for 36 h; DST, leaves after DST for 36 h. Means ± SE of eight replicates are presented. Different letters indicate significant differences between treatments, P<0.05. The differences were analyzed by least significant difference (LSD). (B) Changes of the D1 protein content after high light (HL) and dark-submergence treatment (DST). (C) Quantitative image analysis of protein levels for Fig. 10B. Means ± SE of three replicates are presented. Different letters indicate significant differences between the treatments, P<0.05. Differences were analyzed by least significant difference (LSD).

The activity of PSI complex decreased after 36 h of DST (Fig. 2), which indicates that the PSI activities of leaves were also inhibited during the DST. PSI photoinhibition is usually caused by the degradation of the core protein of PSI, PsaA [32], [33]. Western Blot results showed that PsaA levels dramatically decreased after 36 h of DST (Fig. 5), which demonstrates that DST also damaged the PSI complex.

### Changes of the Chlorophyll and H_2_O_2_ Levels during Dark-submergence Treatment

**Figure 6:**
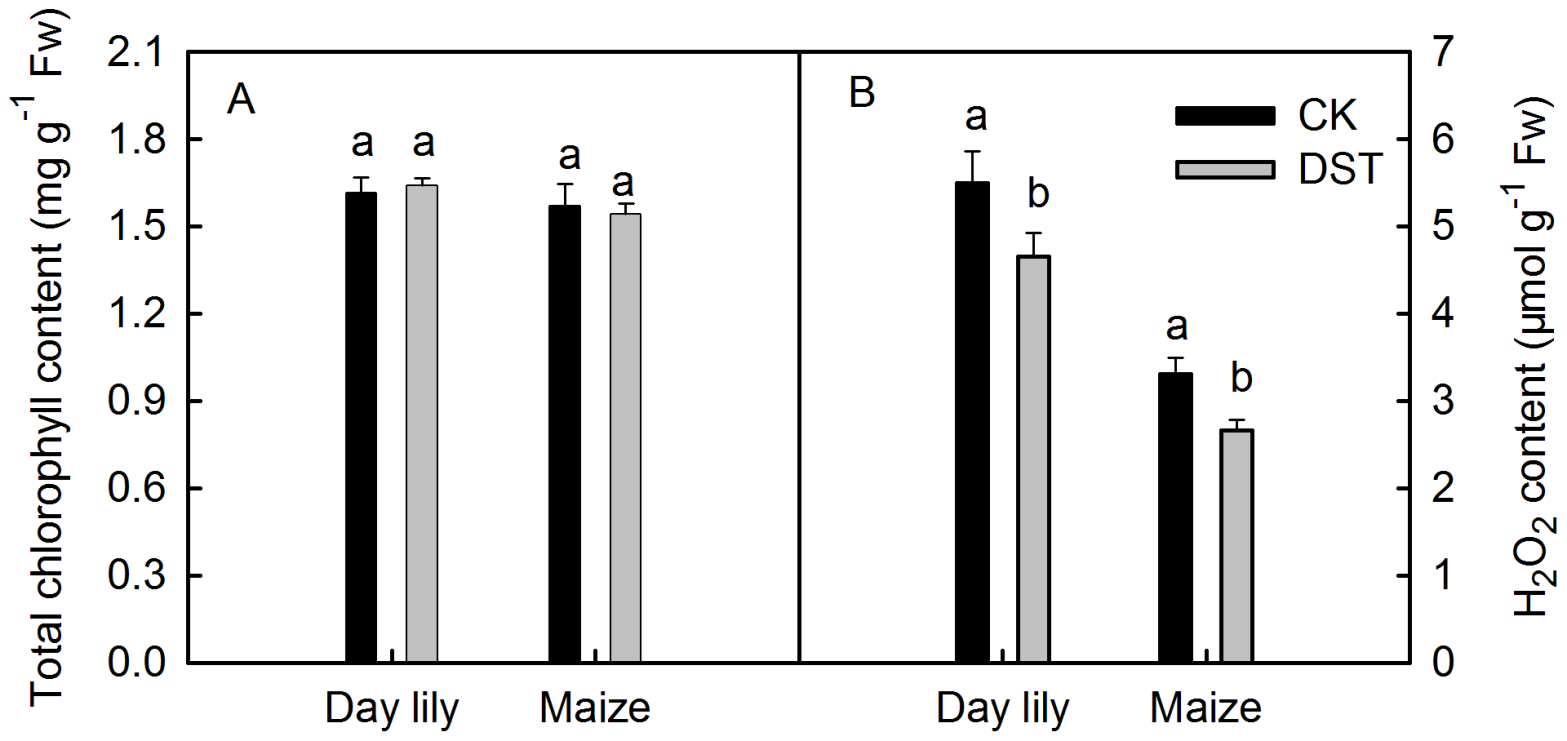
Total chlorophyll and H_2_O_2_ content in leaves before and after dark-submergence treatment. Total chlorophyll (A) and H_2_O_2_ (B) in daylily and maize leaves before and after 36 h of DST. Means ± SE of eight replicates are presented. Different letters indicate significant differences between the different times, P<0.05. Differences were analyzed by least significant difference (LSD).

Most previous studies show that the submergence process is accompanied by leaf senescence [12], [34]. The chlorophyll content, a typical indicator for leaf senescence [35], [36], changed little in leaves of the different species after 36 h of DST (Fig. 6A), which indicates that the decrease of photochemical activity was not induced by leaf senescence.

It has been reported that completely submerged plants produce large amounts of reactive oxygen species (ROS) [37], [38]. Our results showed that the H_2_O_2_ content in different leaves did not significantly increase after 36 h of DST. In contrast, it was lower than that in the control leaves (Fig. 6B), which indicates that the decline of photochemical activity under DST was not due to the over-accumulation of ROS.

### Changes in the Photochemical Activities of Photosystems in Leaves and O_2_ Content in Water during the Dark-submergence Treatment at Different Temperatures

**Figure 7:**
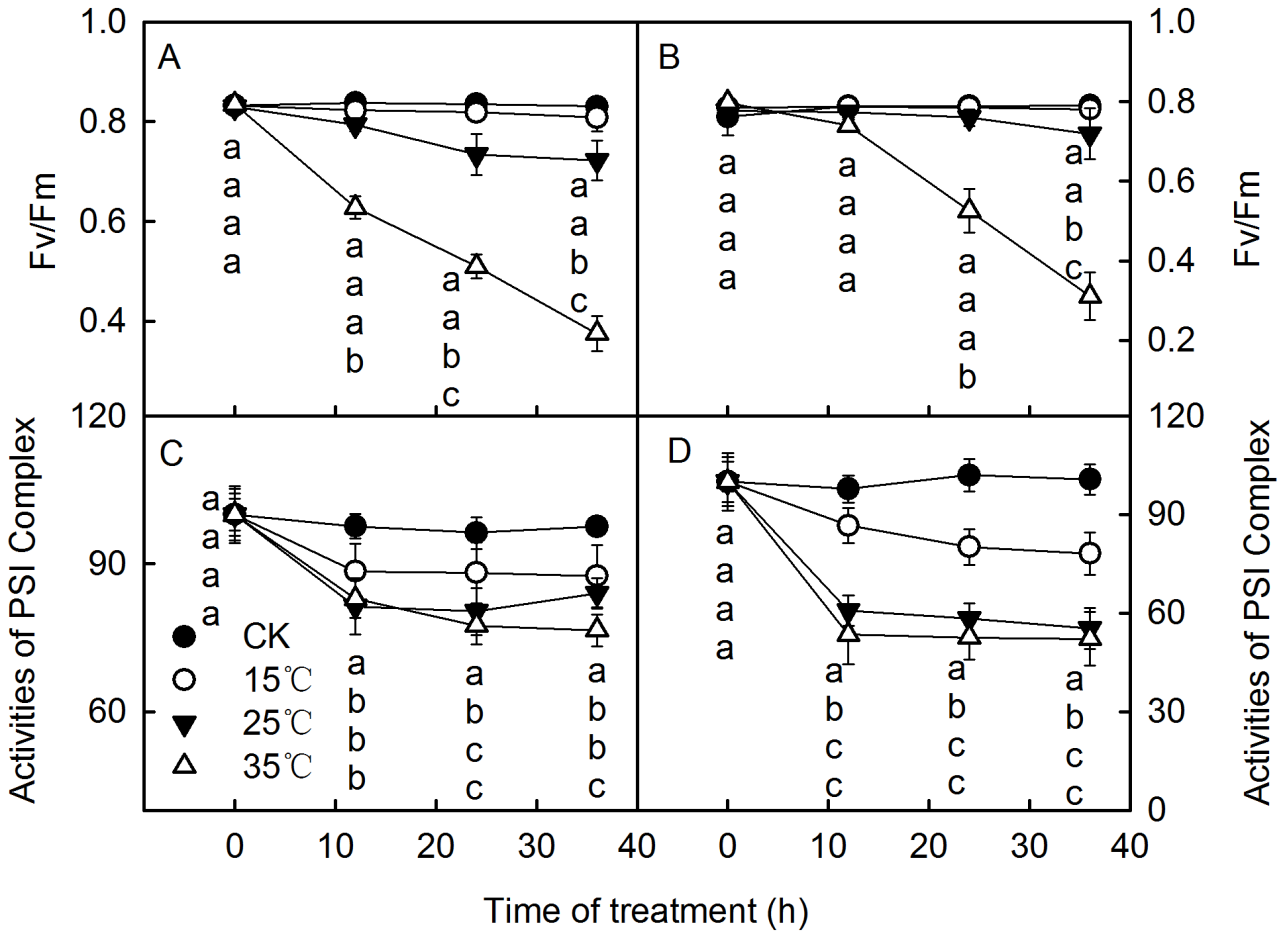
PSII and PSI activity in leaves during dark-submergence treatment at different temperatures. The maximum quantum yield of PSII (Fv/Fm) and activity of PSI complex in leaves of daylily (A, C), and maize (B, D) during dark-submergence treatment (DST) at various temperatures (15°C, 25°C, and 35°C). Means ± SE of eight replicates are presented. Different letters indicate significant differences between the treatments, P<0.05. Differences were analyzed by least significant difference (LSD).

**Figure 8:**
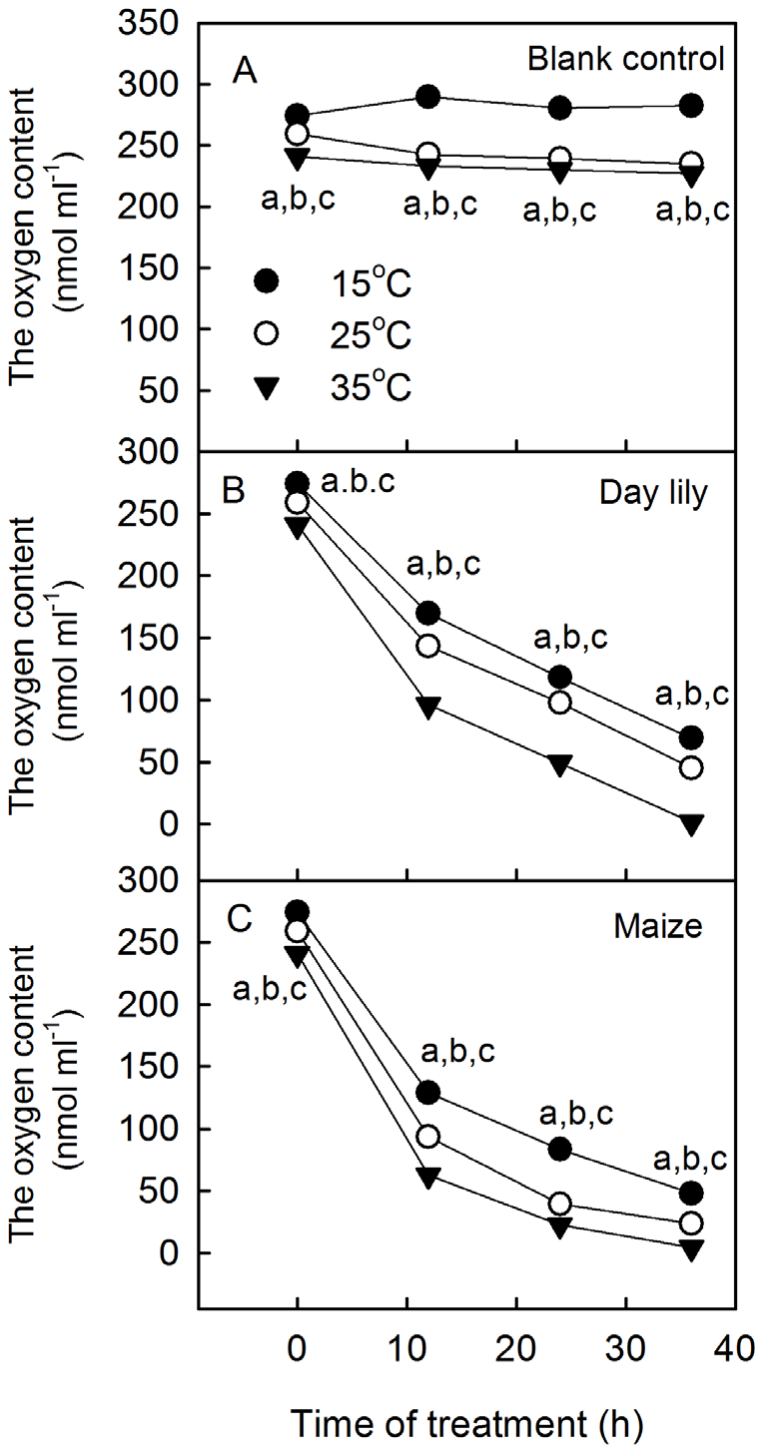
O_2_ content in the water during dark-submergence treatment at different temperatures. The O_2_ content in the water during DST at different temperatures (15°C, 25°C, and 35°C): blank control (A), daylily (B) and maize (C). Means ± SE of three replicates are presented. Different letters indicate significant differences between the various temperatures, P<0.05. Differences were analyzed by least significant difference (LSD).

To further explore the mechanism of the photoinhibition-like damage induced by DST, we submerged leaves in the dark at different temperatures (15°C, 25°, and 35°C). The results showed that the decreases of both Fv/Fm and the activity of the PSI complex were aggravated with the increase of temperature during DST (Fig. 7). In addition, the O_2_ concentrations in the water differed significantly at the various temperatures. The higher the temperature, the lower the O_2_ concentration in water was (Fig. 8). The results above indicate that the photoinhibition-like damage was correlated with the O_2_ concentration in the water. The lower O_2_ level in the water, the severer damage to the photosynthetic apparatus induced by DST was.

### Influence of Air and N_2_ on the Photochemical Activity in Leaves during Dark-submergence Treatment

**Figure 9:**
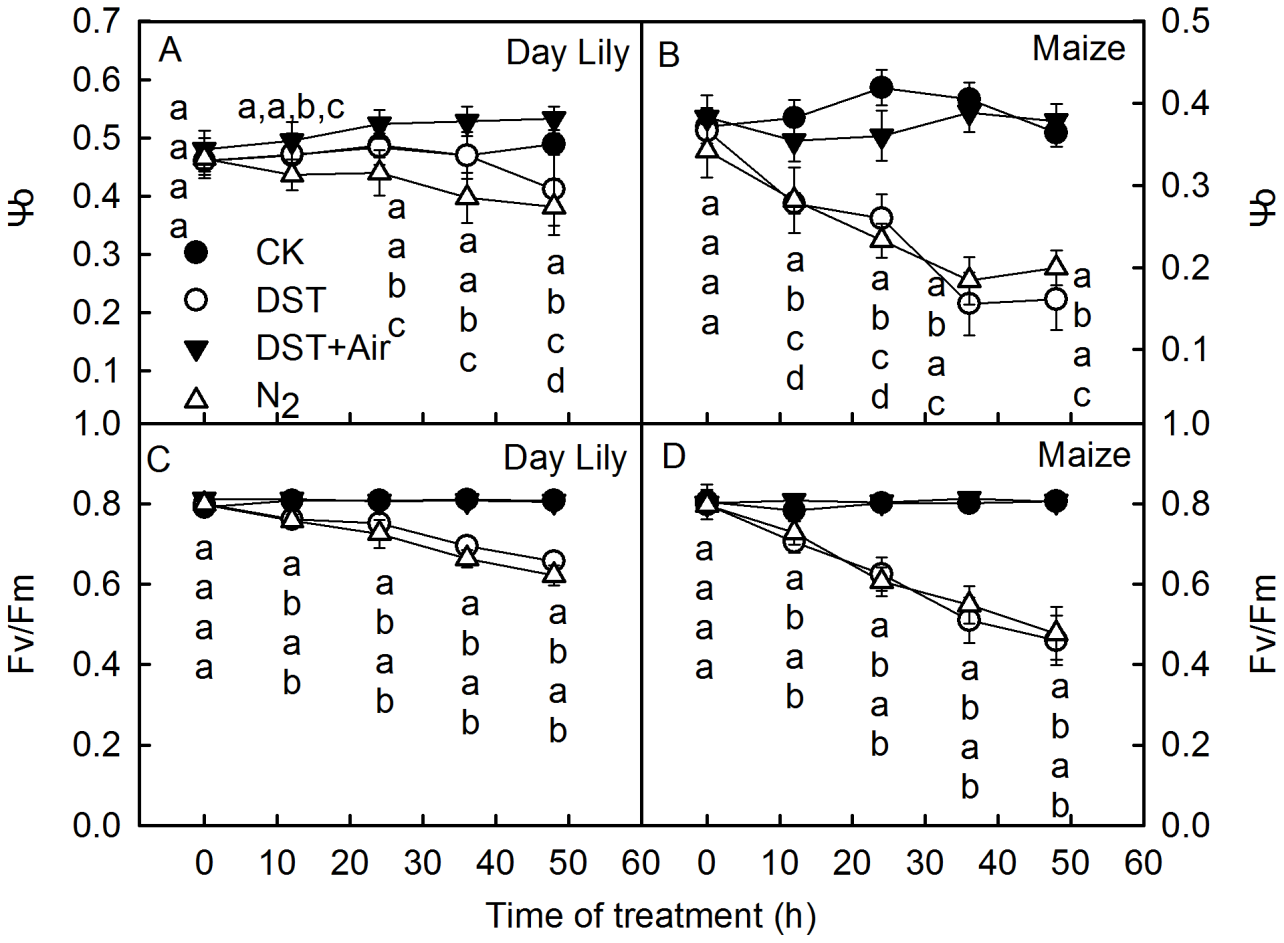
The effect of O_2_ on PSII activity in leaves during dark-submergence treatment. Changes in the efficiency of electron moves beyond Q_A_^−^ (Ψo, A and B) and photochemical efficiency (Fv/Fm, C and D) in daylily (A, C) and maize (B, D) leaves during dark-submergence treatment (DST) or simultaneously pumping air into the water (DST+Air), wrapped in damp cloths and placed in the normal atmosphere (CK) or N_2_ (N_2_). Means ± SE of eight replicates are presented. Different letters indicate significant differences between the treatments, P<0.05. Differences were analyzed by least significant difference (LSD).

To investigate the influence of the O_2_ concentration on leaf photochemical activity, leaves were studied under DST with air pumped into the water (DST+Air). As shown in Fig. 9, no significant changes were observed in Ψo and Fv/Fm between control leaves and leaves treated with DST+Air, which indicates that the decline of photochemical activities in leaves was effectively alleviated or eliminated by the O_2_ supply during DST. In addition, the O_2_ concentration changed little during DST+Air (Table 1). The results above confirm that the O_2_ concentration in the water determined whether the photosynthetic apparatus of leaves would be damaged or remain intact during DST.

**Table 1:** O_2_ concentration in water before and after different treatments.

The O_2_ content in water(nmol ml^−1^)	Day lily	Maize
Before treatment	245.0±0.58^a^	245.0±0.5^a^
After 36 h of DST	49.1±0.71^b^	38.5±1.29^b^
After 36 h of DST+Air	243.0±0.15^a^	225.7±1.00^a^

Changes in the O_2_ concentration of the water before and after pumping air in during dark-submergence treatment (DST). Means ± SE of three replicates are presented. Different letters indicate significant differences between treatments, P<0.05. Differences were analyzed by least significant difference (LSD).

To exclude physical damage of water to the photosynthetic apparatus during DST, we studied the leaves in an artificial hypoxic environment (humid N_2_ atmosphere). The results showed that both the Ψo and Fv/Fm in two plant species significantly decreased after a 36 h treatment under humid N_2_, whereas they remained unchanged after a 36 h treatment with humid air (CK) (Fig. 9). These results further demonstrate that the hypoxic environment plays a crucial role in the damage of the photosynthetic apparatus during DST.

### Influence of Air and N_2_ Supply on the Recovery of Photochemical Activity in Leaves after 36 h of Dark-submergence Treatment

**Figure 10:**
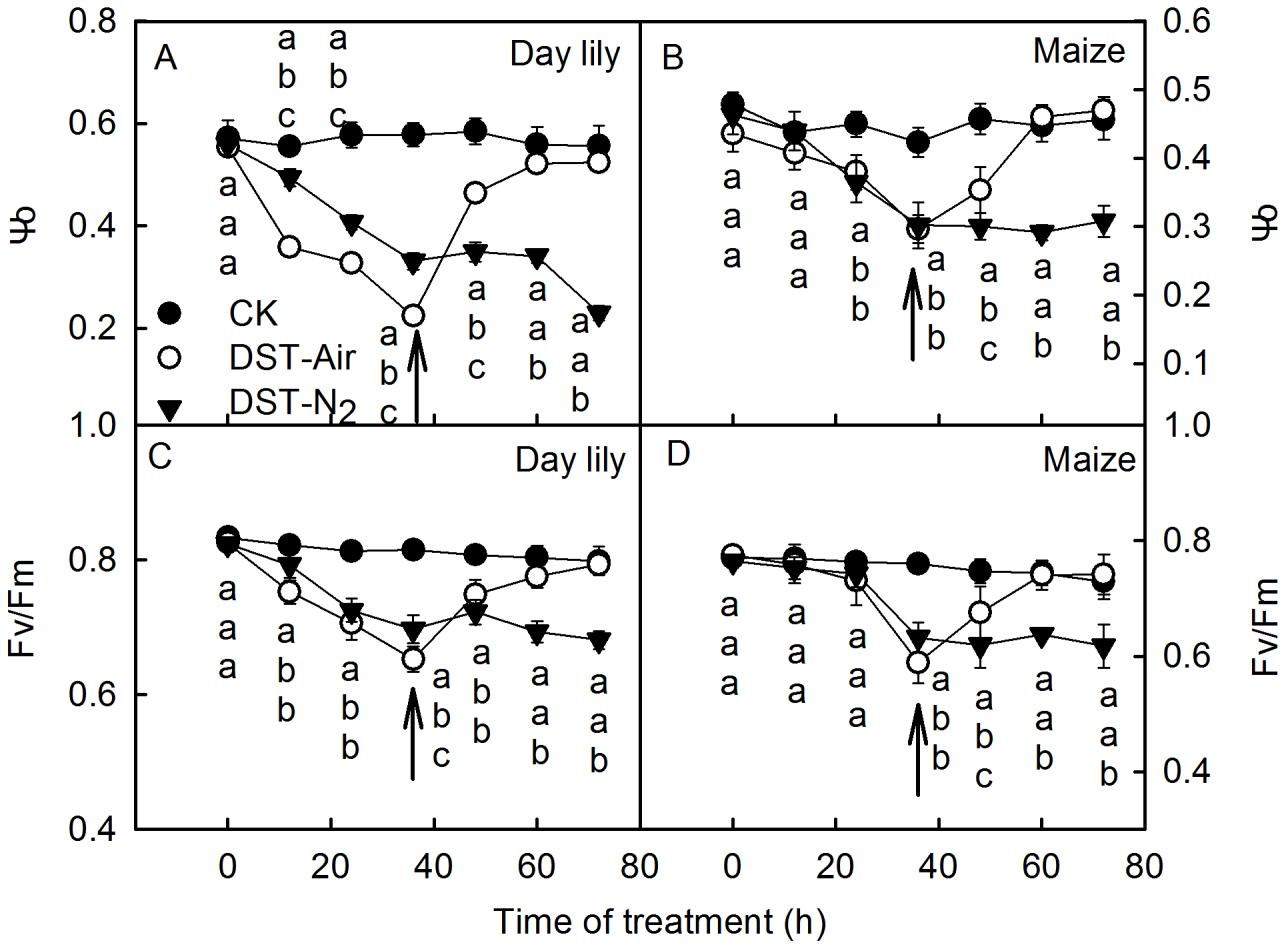
The effect of O_2_ on PSII activity in leaves after dark-submergence treatment. Efficiency of electron moves beyond Q_A_^−^ (Ψo, A and B) and photochemical efficiency (Fv/Fm, C and D) in leaves of the daylily (A, C) and maize (B, D) during dark-submergence treatment (DST) and with air (DST+Air) or N_2_ (DST+N_2_) pumped into the water after 36 h DST. Arrows indicate the time points at which gas was pumped into the water. Means ± SE of eight replicates are presented. Different letters indicate significant differences between the treatments, P<0.05. Differences were analyzed by least significant difference (LSD).

To further investigate the effect of O_2_ on the photosynthetic apparatus after 36 h of DST, air or N_2_ were pumped into the water, and the photochemical activity of leaves was analyzed after 36 h of DST. Results showed that both the Ψo and Fv/Fm recovered to 93.2%∼100% of the control after pumping air into the water after 36 h of DST (Fig. 10). However, both Ψo and Fv/Fm continuously declined after N_2_ was pumped into the water (Fig. 10), which indicates that the damage to the photosynthetic apparatus was effectively recovered by supplying O_2_ after DST. This result further demonstrates that the damage of the photosynthetic apparatus caused by DST is caused by the hypoxic condition of the water.

## Discussion

Most previous studies show that signals from roots and a shortage of CO_2_ in the water may induce leaf senescence and produce excess excitation energy, resulting in decline of the photosynthetic rate and photoinhibition in the leaves of submerged plants. However, in this study, we observed that the activity of the photosynthetic apparatus was inhibited during DST in detached leaves. This inhibition was independent of root signals and excess excitation energy.

DST significantly damaged the donor side of PSII, which was indicated by a significant increase of the K step in the OJIP transients (Fig. 1) and the relative variable fluorescence at the K step (W_K_) (Fig. 3), the effective indicators of injury to the donor side of PSII [24], [27], [28]. The dramatic decline of the O_2_ evolution capacity of DST leaves (Fig. 3) further demonstrates that the donor side of PSII was damaged during DST. DST also inhibited the acceptor side of PSII, which was shown by an increase of the J steps in the OJIP transients (Fig. 1) and a decline in the efficiency of electron moves beyond Q_A_^−^ (Ψo) (Fig. 2). In addition, the reaction centers of PSII were remarkably damaged by DST, which was reflected by a decrease in the maximum quantum yield of PSII (Fv/Fm) and the density of Q_A_-reducing PSII reaction centers per cross section (RC/CSm). The above results demonstrate that the activities of the donor and acceptor sides and reaction center were inhibited during DST. The decrease of Fv/Fm, RC/CSm and Ψo are conventional indicators of photoinhibition [29]–[31]. However, we observed similar damage to the photosynthetic apparatus without light in different plant species. Therefore, we refer to this DST-induced damage as “photoinhibition-like damage”.

The traditional light-induced photoinhibition is due to net degradation of D1 protein [29], [30], [39]. In this study, the decrease in Fv/Fm was accompanied by a significant increase in J steps in the OJIP transients (Fig. 1) and a decline in Ψo (Fig. 2) during DST, which indicates that the electron transport from Q_A_ to Q_B_ was inhibited by DST [24], [26]. D1 is a core protein of the PSII reaction center, which is associated with Q_B_. The inhibition of electron transport from Q_A_ to Q_B_ may consequently be related to the degradation of the D1 protein. Through Western Blot analysis, we observed that D1 was significantly degraded by DST (Fig. 4). This confirmed that photoinhibition-like damage by DST was partly caused by the degradation of D1.

The PSI activity was also inhibited during DST, as illustrated by the significant decrease in activity of the PSI complex (Fig. 2). The finding that levels of PSI core protein PsaA significantly decreased during DST (Fig. 5) further supported this conclusion.

Some previous studies showed that, during submergence of a whole plant, roots under hypoxic conditions may produce signal substances [12], [34]. This includes ethylene, which is transported to shoots leading to leaf senescence, characterized by chlorophyll degradation [35], [36]. It has been reported that ROS are over-accumulated in submerged plants [37]. During submergence in the light, more photosynthetic electrons will be transported to O_2_ to produce ROS [37], [38], due to the over-reduction of the PSI acceptor side. However, in this study, we observed no significant decrease in chlorophyll content in leaves during DST (Fig. 6A), which indicates that the associated damage to the photosynthetic apparatus was not caused by leaf senescence. No significant increase in H_2_O_2_ was observed in leaves after DST (Fig. 6B), indicating that the photoinhibition-like damage was not caused by the over-accumulation of ROS.

DST resulted in hypoxia, since the O_2_ concentration in water decreased to 15.7∼20% after 36 h (Table. 1). Hypoxia might be a cause of the photoinhibition-like damage. However, it is unclear whether a hypoxic environment can damage the photosynthetic apparatus in the dark. To clarify the role of hypoxia on the photosynthetic apparatus damage of plant leaves, we analyzed the photochemical activity of leaves under DST at different O_2_ concentrations. This confirmed that the hypoxic environment caused the photoinhibition-like damage in leaves under DST. This was supported by the following results: 1) Supplying air to the water almost completely eliminated the damage (Fig. 9). 2) When the damage occurred in leaves under DST, photochemical activities of leaves almost completely recovered when air was supplied to the water (Fig. 10). 3) Placing leaves in a humid N_2_ condition in the dark caused a similar decline of photochemical activity, as did DST (Fig. 9). 4) The damage to the photosynthetic apparatus in leaves was aggravated with increasing temperatures (15°C, 25°C, and 35°C) (Fig. 7), which enhanced O_2_ consumption by higher respiration of leaves (Fig. 8).

The hypoxic environment decreased the photosynthetic activity of leaves in the dark, which might be caused by anaerobic respiration products such as ethanol to the photosynthetic apparatus. In addition, the decline of the ATP supply during anaerobic respiration may limit the synthesis of proteins relevant to the photosynthetic apparatus, such as D1 and PsaA. This is supported by the finding that D1 and PsaA levels significantly decreased during DST (Fig. 5).

In conclusion, DST caused photoinhibition-like damage to the photosynthetic apparatus, independent of light and root signals. The photoinhibition-like damage was caused by the hypoxic environment during DST, which inhibited the synthesis of core proteins of the photosynthetic apparatus, such as D1 and PsaA. Further studies are needed to explore the detailed mechanism by which the hypoxic environment damages the photosynthetic apparatus.
